# Twelve-Lead Electrocardiogram Acquisition With a Patchy-Type Wireless Device in Ambulance Transport: Simulation-Based Randomized Controlled Trial

**DOI:** 10.2196/24142

**Published:** 2021-04-01

**Authors:** Sunyoung Yoon, Taerim Kim, Taehwan Roh, Hansol Chang, Sung Yeon Hwang, Hee Yoon, Tae Gun Shin, Min Seob Sim, Ik Joon Jo, Won Chul Cha

**Affiliations:** 1 Department of Digital Health Samsung Advanced Institute for Health Science & Technology (SAIHST) Sungkyunkwan University Seoul Republic of Korea; 2 Department of Emergency Medicine Samsung Medical Center Sungkyunkwan University School of Medicine Seoul Republic of Korea; 3 Healthrian Co, Ltd Dajeon Republic of Korea; 4 Health Information and Strategy Center Samsung Medical Center Seoul Republic of Korea

**Keywords:** 12-lead electrocardiogram, electrocardiogram transmission, prehospital, wearable patch device, wearable, electrocardiogram, ECG, cardiovascular, efficiency, feasibility, EMT

## Abstract

**Background:**

Cardiovascular disease is the leading cause of death worldwide. Early recognition, diagnosis, and reperfusion are the key elements of treatment for ST-segment elevation myocardial infarction. The absence of a prehospital 12-lead electrocardiogram (P12ECG) can cause definitive treatment delay and repeated transfer. Although guidelines highly recommend the measurement and transmission of P12ECG data, P12ECG use has not been widely established.

**Objective:**

The aim of this study was to verify the time-efficiency and feasibility of the use of a patchy-type 12-lead ECG measuring and transmitting device (P-ECG) by an emergency medical technician (EMT) in an ambulance during patient transport.

**Methods:**

This was a simulation-based prospective randomized crossover-controlled study that included EMTs. The participants were randomly assigned to one of two groups. Group A began the experiment with a conventional 12-lead ECG (C-ECG) device and then switched to the intervention device (P-ECG), whereas group B began the experiment with the P-ECG and then switched to the C-ECG. All simulations were performed inside an ambulance driving at 30 km/h. The time interval was measured from the beginning of ECG application to completion of sending the results. After the simulation, participants were administered the System Usability Scale questionnaire about usability of the P-ECG.

**Results:**

A total of 18 EMTs were recruited for this study with a median age of 35 years. The overall interval time for the C-ECG was 254 seconds (IQR 247-270), whereas the overall interval time for the P-ECG was 130 seconds (IQR 112-150), with a significant difference (*P*<.001). Significant differences between the C-ECG and P-ECG were identified at all time intervals, in which the P-ECG device was significantly faster in all intervals, except for the preparation interval in which the C-ECG was faster (*P*=.03).

**Conclusions:**

Performance of 12-lead ECG examination and transmission of the results using P-ECG are faster than those of C-ECG during ambulance transport. With the additional time afforded, EMTs can provide more care to patients and transport patients more rapidly, which may help reduce the symptoms-to-balloon time for patients with acute coronary syndrome.

**Trial Registration:**

ClinicalTrials.gov NCT04114760; https://www.clinicaltrials.gov/ct2/show/NCT04114760

## Introduction

### Background and Significance

Cardiovascular disease (CVD) is the leading cause of death worldwide [1]. In 2017, there were 17.9 million deaths resulting from CVD, more than three-quarters of which occurred in low- or middle-income countries [2]. Deaths caused by ischemic heart disease have risen by about 19%-20% over the past 10 years. Although age-standardized death rates from CVD have shown a decreasing trend globally, CVD remains one of the primary causes of death [3]. To reduce the total ischemic time, as a major factor in short- and long-term mortality [1,4,5], early recognition, diagnosis, and reperfusion should be performed in a coordinated and complementary manner.

### Prehospital 12-Lead Electrocardiogram

The prehospital 12-lead electrocardiogram (P12ECG) is a cornerstone of emergency cardiac care. Capturing and transmitting P12ECG data to the emergency department is an integral part of patient care for acute coronary syndrome (ACS) [6], and serves as an inexpensive, noninvasive diagnostic tool. Both the American Heart Association and the European Society of Cardiology recommend the capture and transmission of P12ECG data for patients who present with symptoms suggestive of ACS [1,4]. Missing P12ECG in patients with suspected ACS is a risk factor for delay in reperfusion treatment or transfer to a nondesignated hospital [7].

However, use of P12ECG has not been widely established. A large retrospective cohort study performed in the United States found that before hospital arrival, only 32% of patients had received P12ECG, and among the patients who visited the emergency department via a 911 call with a final confirmed diagnosis of ACS, almost 41% had not received prehospital electrocardiogram (ECG) [8]. Other studies have also indicated that P12ECG data are transmitted to the hospital for only a small portion of patients. A study performed in Los Angeles found that among those diagnosed with ST-segment elevation myocardial infarction (STEMI) by P12ECG, only 28% had their data transmitted to the hospital; in 55% of cases, the data were not transmitted and for 17% of these cases the transmission status was not recorded [9]. In another study performed in Poland, 12-lead ECG was transmitted to the hospital by an emergency medical service (EMS) team for only 2% of patients [10].

Identifiable reasons for missing P12ECG include transmission malfunction, too short transfer interval for performing P12ECG, and only a 3-lead ECG available [11]. The most frequently used 12-lead ECG examination device during the prehospital stage and in the ambulance is the defibrillator; however, this device can transfer ECG data only by registered email [12]. Various handheld and wearable ECG devices [13,14] and transmission technologies [15-19] have been developed in recent years, but few studies have examined both the device application and transmission of P12ECG in an ambulance during patient transport.

### Study Objective

The aim of this study was to verify the time-efficiency and feasibility of the use of a patchy-type 12-lead ECG measuring and transmitting device (P-ECG) by emergency medical technicians (EMTs) in an ambulance during patient transport.

## Methods

### Study Design

This was a simulation-based prospective randomized crossover-controlled study that included EMTs. The experiment was performed in an ambulance driving 30 km/h. The experiment was performed at a single institution and the driving course was limited to the hospital site. This study used a case-crossover design and participants were randomly assigned to one of two groups.

This study was approved by the Samsung Medical Center Institutional Review Board (No. 2019-04-004) and is registered at ClinicalTrials.gov (NCT04114760).

### Study Setting

The EMS system in Korea consists of 18 provincial headquarters, 224 fire stations, and 1474 ambulance stations. There are 12,033 EMTs registered in the system who are classified into level 1 or level 2 certification, which determines their scope. The scope of procedures and skills corresponding to level-1 EMTs in Korea are comparable to those of EMTs at the intermediate level in North America. Intermediate/level-1 EMTs can perform a 12-lead ECG examination with physician oversight.

### Study Population and Sample Size

The inclusion criteria were as follows: aged 19 years or older and a level-1 certified EMT. The exclusion criteria were as follows: not working as a field agent (eg, a dispatcher) and only a level-2 EMT license. Study recruitment posters were posted in a fire station in Seoul, and participants were recruited through voluntary participation. All participants were given a reward worth approximately US $20.

The sample size was calculated based on our unpublished pilot study involving hospital health care providers. Participants were divided into two groups: the conventional 12-lead ECG (C-ECG) group and the P-ECG group. The mean time required in the C-ECG group was 161.35 seconds (SD 81.57) and the mean time required in the P-ECG group was 79.12 seconds (SD 24.75). The Wilcoxon signed-rank test was used to compare the means between paired samples with a crossover design. The minimum number of samples needed for hypothesis testing was calculated with a power of 0.95, effect size of 1.13, and type I error of .05. The effect size was estimated based on previous studies and calculated using G power 3.1.9.4. [20]. Based on the above information, it was determined that the minimum number of participants needed for this study was 13. Assuming a 40% dropout rate, 18 was the target number of participants.

Data on the demographic and study-related characteristics of the participants were collected, including gender and age, previous hospital training, and number of years as an EMT. In addition, education and training were provided to the participants by a professor of emergency medicine. The training included instruction on proper 12-lead ECG methods, introduction to the P-ECG, practice, and a question and answer session.

### Materials

**Figure 1:**
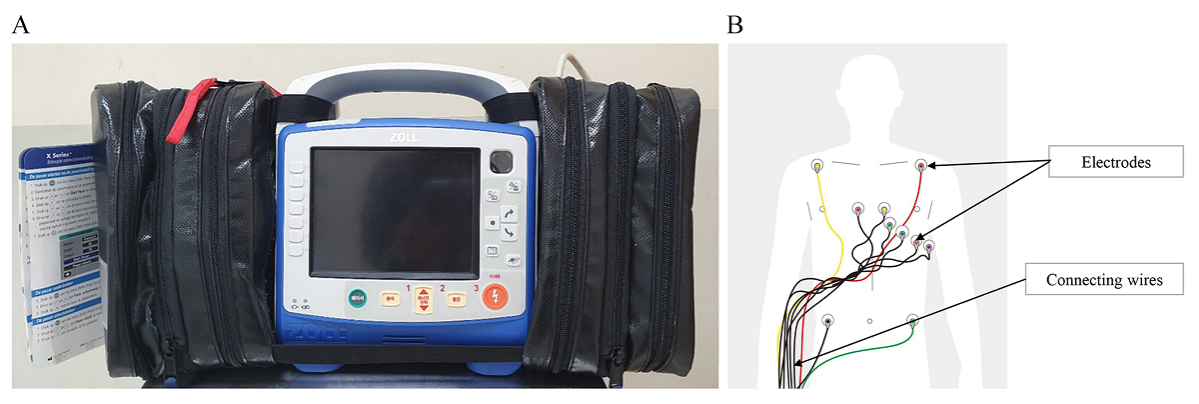
(A) The conventional 12-lead electrocardiogram device used for the control condition (X-series, ZOLL Medical, Chelmsford, MA, USA). (B) Attachment of electrodes on the patient's body surface, which are connected to the main device.

The ZOLL X series defibrillator (Chelmsford, MA, USA) was used as the C-ECG device and wearECG12 (HEALTHRIAN, Daejeon, Republic of Korea) was used as the P-ECG device. The X-series is 22.6x×26.4×20.1 cm and weighs 5.3 kg (including the battery and paper). The X-series is currently the most commonly used equipment in the Korean EMS system and is included among the first-aid equipment mounted in the ambulance. The device is capable of both defibrillation and ECG monitoring. A mode setting, cable change, and 10-electrode attachment are required for the 12-lead ECG examination, and the results are printed on paper (Figure 1).

**Figure 2:**
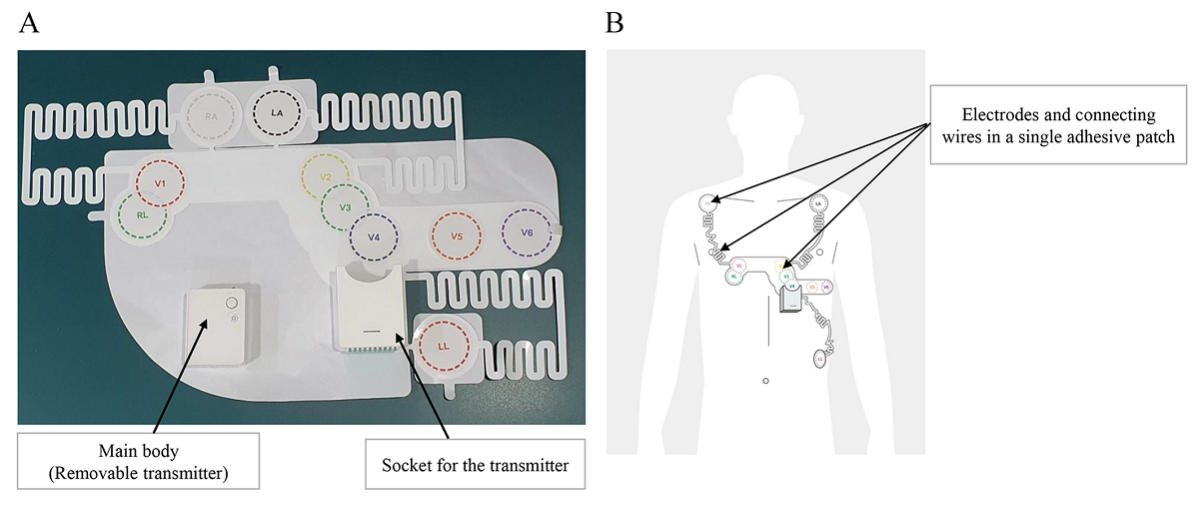
(A) Patchy-type wireless 12-lead electrocardiogram used for the intervention condition (HEALTHRIAN, Yuseong-gu, Daejeon, Republic of Korea). The device consists of two parts: a flexible patch with a socket and a transmitter. (B) Application of the device with the transmitter on the patient’s left chest area.

The P-ECG, which was used as the intervention device, was approved by the Korean Ministry of Food and Drug Administration as Holter ECG technology (Figure 2). This device consists of a main body (46×35.6×16 mm, 30 g) and a one-patch type electrode (241.19×375.5 mm, 35 g). The main body is assembled on the socket of the patch. For performing 12-lead ECG examinations, the tablet and main body are wirelessly connected via Bluetooth (Kirkland, WA, USA) and provide continuous monitoring. The results of an ECG examination are generated in a PDF that was transmitted in real time to the researchers’ dashboards via long-term evolution (LTE) networks when the tablet’s “upload” button was clicked. The tablet used in this study was Samsung Galaxy Tab S3 (SM-T825; Seoul, Republic of Korea) on an LTE network. Samsung Galaxy Books (SM-W627NZFKOO) was used as the dashboard on an LTE network. The differences between the two ECG devices are summarized in Table 1.

**Table 1:** Functional comparison between the conventional 12-lead electrocardiogram (C-ECG) and patchy-type wireless 12-lead electrocardiogram (P-ECG) devices.

Component	C-ECG	P-ECG
Electrode	10 electrodes (separated)	Single electrode (combined as a patch)
Wire	10 wires for each electrode	Wireless (conductible film on the patch)
Control	Manual control (minimum 10 steps)	Semiautomatic (4 steps)
Transmission	Messenger app (device supports email with a cellular dongle)	Built-in app

### Study Protocol

**Figure 3:**
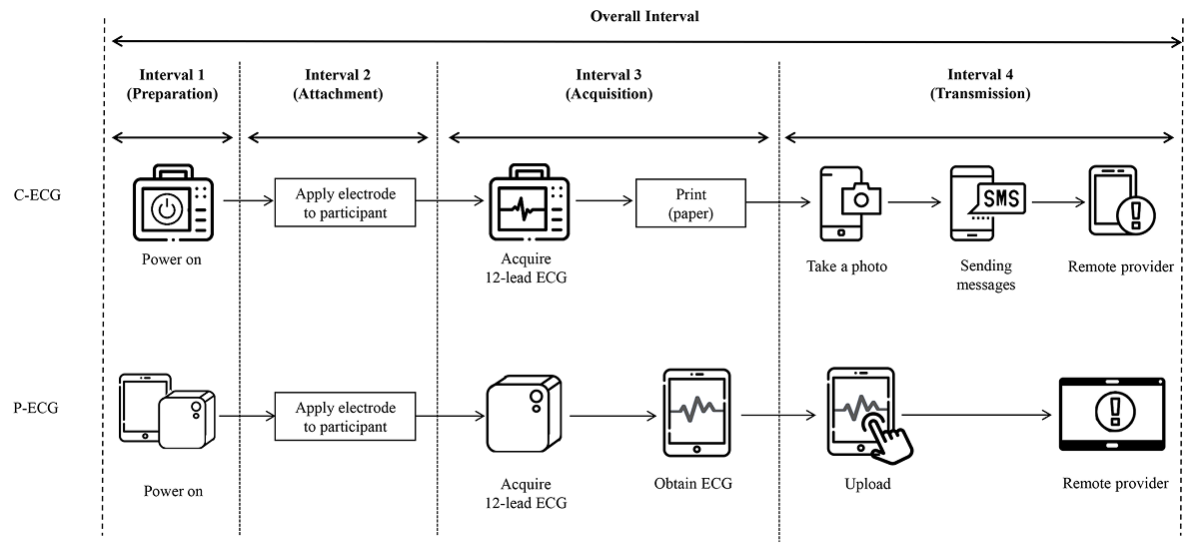
Definition of outcomes (intervals). The overall interval was defined as the interval from the "start" command to the acquisition of an electrocardiogram (ECG) image by a remote provider. C-ECG: conventional electrocardiogram; P-ECG: patchy-type electrocardiogram.

All participants provided written informed consent before the start of the simulation. They were provided with 15 minutes of education on 12-lead ECG and the two devices, followed by 10 minutes of practice. All participants already knew how to use the C-ECG and therefore only practiced using the P-ECG. They were randomly assigned to one of two groups. Group A began the experiment with the C-ECG and then transferred to the P-ECG, whereas group B began the experiment with the P-ECG and then transferred to the C-ECG. Both groups performed the 12-lead ECG examination on a simulated mock patient. The volunteers were selected according to the inclusion criteria of healthy men aged 19 years or older. Six healthy men aged 28 to 35 years were included as mock patients. Each mock patient received an ECG examination six times by the EMT participants. Testing was performed in the same order for both devices: preparation, attachment, acquisition, and transmission (Figure 3).

The following procedure was used for the C-ECG (Figure 3): (1) preparation, turn on the device and change the mode and cable to that for 12-lead ECG; (2) attachment, apply the 10 electrodes and the wire cable, and modify the positions of the electrodes as needed so that the four limb leads are located on both the upper shoulder and lower abdomen (Figure 1B); (3) acquisition, perform the 12-lead ECG examination and print the results; (4) transmission, take a photo of the printout of the results and send it in a message to the researcher’s cell phone.

The procedure for the P-ECG was as follows (Figure 3): (1) preparation, turn on the main body of the device, assemble it on the patch’s socket, and complete Bluetooth pairing; (2) attachment, apply the one-patch type electrodes and modify the electrode positions as needed (Figure 2B); (3) acquisition, perform the 12-lead ECG examination and obtain the ECG results (PDF); (4) transmission, complete the transmission of the results to the researcher’s dashboard using the LTE network by touching the “upload” button on the tablet.

**Figure 4:**
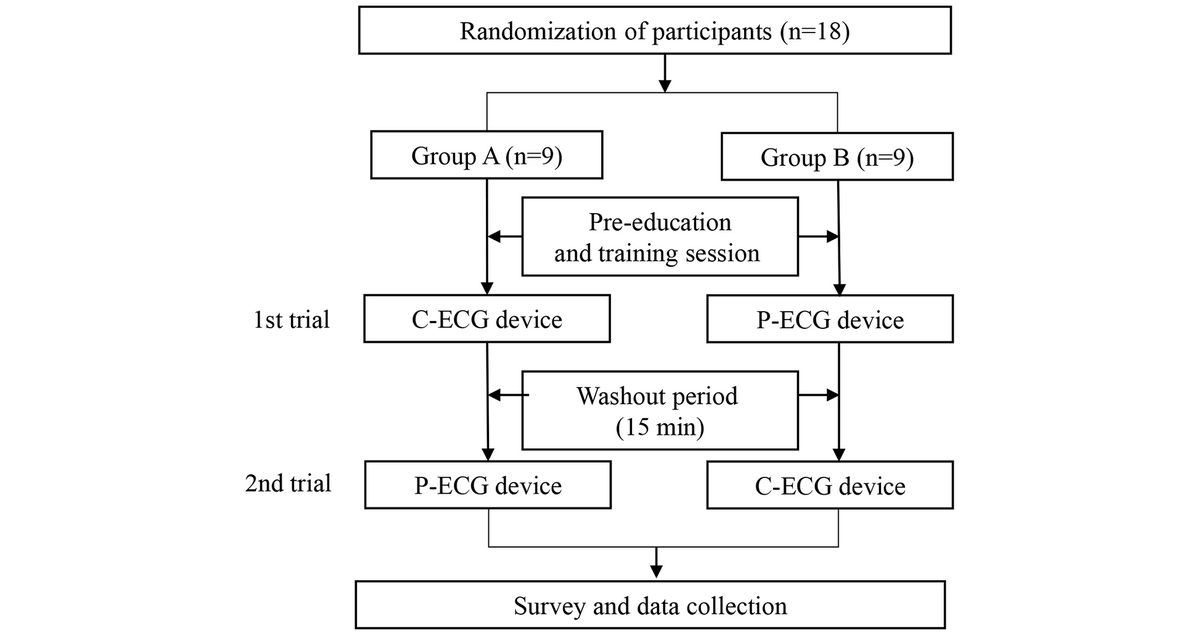
Case-crossover design study process. There was a washout period before two trials. C-ECG: conventional 12-lead electrocardiogram; P-ECG: patchy-type wireless 12-lead electrocardiogram.

After the simulations, a modified version of the System Usability Scale (SUS) [21] was administered to all participants to assess their satisfaction with the patchy-type wireless device (Figure 4).

### Outcome Measures

The primary outcomes of the study were the time intervals during each stage (Figure 3). Interval 1 was the preparation time, from powering on to having the system ready for ECG acquisition. Interval 2 was the time for attachment, measured from the beginning of electrode attachment to final modification of the position of the attached electrodes. Interval 3 was the time taken to acquire a complete 12-lead ECG for transmission. Interval 4 was the time for transmission, from completion of 12-lead ECG results acquisition to completion of transmission to a distant health care provider. The overall interval was calculated as the sum of intervals 1 to 4.

All steps were compared and verified using a stopwatch and video recording to ensure time interval accuracy. The video recording did not reveal the faces of the participants, and as stated on the consent form, the video was only used for research purposes.

The secondary outcome of the study was the SUS score. This outcome was used to investigate participant satisfaction with the P-ECG device. In this study, the SUS was administered only for the P-ECG. There were two reasons for assessing the usability of only one device. First, this study was designed as a case-crossover study. All participants used both devices and the order of experience was randomized. Having mixed experiences could have made it difficult for participants to clearly respond to two surveys at the same time. Second, all participants were already familiar with the C-ECG, which is among the required first-aid equipment in the ambulance. Therefore, the score for the conventional device would likely have been much higher regardless of the usability of the device itself. The SUS consists of 10 questions, each based on a 5-point Likert scale ranging from 1 (“strongly disagree”) to 5 (“strongly agree”). The formula used to calculate the SUS score was as follows [21]:

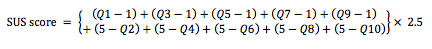



The SUS score was evaluated based on a previous study [22], which interpreted mean SUS scores above 12.5 as “Worst Imaginable,” above 20.3 as “Awful,” above 35.7 as “Poor,” above 50.9 as “OK,” above 71.4 as “Good,” above 85.5 as “Excellent,” and above 90.9 as “Best imaginable.” 

### Data Analysis

The differences in paired values of the time required for the same participant were analyzed using the Wilcoxon signed-rank test. *P* values less than .05 were considered statistically significant. All data processing and statistical analyses were performed using R version 3.6.3 software (R Foundation for Statistical Computing, Vienna, Austria).

## Results

### Participant Characteristics

Table 2 presents the demographic and study-related characteristics of the participants. The median age of the participants was 35 years. The median continuous years of service as an EMT was 8 years and 5 months (IQR 4 years, 7 months to 13 years, 1 month). About half of all participants had worked in a tertiary hospital for more than 2 years before serving in the EMT (Table 2).

**Table 2:** Demographic and study-related characteristics of the participants (N=18).

Characteristic		Value
**Gender, n (%)**		
	Women	4 (22)
	Men	14 (78)
Age (years), median (IQR)		35 (32-42)
**Previous training in the hospital, n (%)**		
	Yes	10 (56)
	No	8 (44)
**Years of service in the emergency medical service, n (%)**		
	0-4	4 (22)
	5-8	5 (28)
	9-18	9 (50)

### Time Interval Comparison

The overall interval time for the C-ECG device was significantly slower than that for the P-ECG device (Table 3). Significant differences between the C-ECG device and the P-ECG device were identified at all time intervals, in which the P-ECG device was significantly faster for all intervals except interval 1 (Table 3).

**Table 3:** Comparison of time intervals between the conventional electrocardiogram (C-ECG) and patchy-type electrocardiogram (P-ECG) devices.

Interval	C-ECG, median (IQR)	P-ECG, median (IQR)	*P* value^a^
Interval 1 (preparation)	26 (22-33)	34 (31-47)	.03
Interval 2 (attachment)	77 (64-91)	53 (43-75)	.03
Interval 3 (acquisition)	69 (66-75)	24 (21-30)	<.001
Interval 4 (transmission)	74 (58-97)	6 (4-8)	<.001
Overall interval	254 (247-270)	130 (112-150)	<.001

^a^*P* values were calculated using the Wilcoxon signed-rank test.

### SUS Survey Outcomes

According to a previous study, SUS scores higher than 71.4 can be interpreted as “good” [22]. In this study, the total SUS score was 73.75, indicating that the P-ECG has good usability and is acceptable to the user (Table 4).

**Table 4:** System Usability Scale adapted for assessment of participant satisfaction with the patchy-type wireless electrocardiogram (ECG) device.

Question	Mean (SD)^a^
I think that I would like to use the ECG device frequently	3.33 (0.91)
I found the ECG device to be unnecessarily complex	2.50 (1.15)
I thought the ECG device was easy to use	3.33 (0.84)
I think that I would need the support of a technical person to be able to use the ECG device	2.39 (1.33)
I found that the various functions in the ECG device were well-integrated	2.78 (0.94)
I thought there was too much inconsistency in the ECG device	3.00 (0.69)
I would imagine that most people would learn to use the ECG device very quickly	3.50 (0.79)
I found the ECG device very cumbersome to use	3.22 (1.11)
I felt very confident using the ECG device	3.06 (0.64)
I need to learn a lot of things before I could get going with the ECG device	2.39 (1.14)
Total score	73.75 (17.58)

^a^Scored on a scale from 1 (“strongly disagree”) to 5 (“strongly agree”).

## Discussion

### Principal Results

To the best of our knowledge, our study is the first to investigate a new P12ECG device and system using an LTE network and a single patchy-type 12-lead ECG, transmitting real-time 12-lead ECG data to dispatcher dashboards during ambulance transport. Except for during the preparation stage, the intervention device was at least 20 seconds faster during all stages and was approximately 70 seconds faster during the transmission stage. As a result, the total time from preparation to transmission completion was reduced from 4 minutes to 2 minutes, despite this being the first use of an unfamiliar device.

Although a 2-minute difference may seem trivial, this corresponds to nearly half of the conventional mean. With that additional time, EMTs can provide much needed care to the patient and transport the patient more rapidly to the destination hospital. Although it was not determined in this study, the new system requires very little interaction once attached, and the 2 minutes of application time required for the new device may be reduced given the EMT’s free hands.

A prior study found that the main reason for failure of performed or transmitted P12ECG was a short transfer time to the hospital [11], suggesting that reducing the total required time of P12ECG by half would improve the P12ECG measurement and transmission rate within 10 minutes of first medical contact. In addition, reducing 2 minutes in the prehospital field is more critical than in the in-hospital setting because delays in testing can result in delays in transport and definitive care.

### Downsides of the New Device

There has been a relative decline in ACS mortality trends in all national regions; however, the prevalence rates differ depending on the country’s gross domestic product per capita [23]. ACS mortality trends are also influenced by various factors such as the patient’s medical condition (eg, the presence of diabetes mellitus, renal failure, or previous coronary artery disease) and performance of the STEMI network based on the emergency medical and primary health care system [1]. Although P12ECG transmission is considered a cornerstone, the cost and necessary regional infrastructure to maintain such activity could be a burden in many regions.

Although not proven in this study, there could be two major challenges to the wide adoption of P12ECG. The first is the price. Conventional ECG uses very cheap, disposable electrodes, and the new device’s patch includes electrodes and wire-like films, which are also disposable, making its use more expensive. The second is the placement of electrodes on the surface of the body; the new device’s lib leads are placed in a more proximal area compared with conventional devices, which makes it difficult to compare ECG readings from different ECG types.

### Potential of the New Device

In the emergency medical system, particularly during the prehospital stage, an EMS-friendly device is required. ECG technology was developed about 120 years ago by Einthoven. The initial model required patients to place their extremities in buckets for measurement. The present 12-lead ECG was invented about 80 years ago by Dr. Emanuel [24] and is only used inside of a hospital in early states. Although some studies have found the prehospital and in-hospital quality of 12-lead ECG to be equal [25-28], it is difficult to actively apply large and heavy defibrillator devices in the prehospital environment. Most potential ACS patients are alert and rarely need defibrillation immediately; therefore, the advantage of separating a 12-lead ECG device from the defibrillator is expected to increase the use of P12ECG.

Well-planned and well-coordinated programs that provide sufficient training are essential for the effective implementation of P12ECG [29]. During the 15-minute pretraining session used in our study, most participants took some time to get used to the new device and system. The lack of sufficient training time could increase the total time as the primary outcome of this study. The SUS results suggest that this might have been the case. The question with the highest rate of agreement was related to believing that most people would learn to use the ECG device very quickly, whereas the questions related to evaluating device function as being well-integrated and feeling confident using the ECG device had the highest rate of disagreement. That is, participants believed they could learn to use the device quickly but they were not yet familiar with the new device function and were not confident after the short training they received for the study. Despite the short training time and low confidence, the overall time was reduced by nearly half in this study, and it is highly likely that more familiarity with P-ECG, leading to equivalent confidence in using C-ECG, would increase the difference in the overall time interval, eventually making the outcome more convincing.

Current technology offers a more enhanced user experience and is more usable compared with previous devices. A novel, smart device shortens the learning period through its simpler and more intuitive design. Although the use of new devices and systems may initially cause discomfort and lower confidence, there may be sufficient improvement with training.

### Optimized Networking System for Hardware and Software

Easy and fast transmission methods are also essential in applying P12ECG. Because real-time decision-making is needed, EMTs share ECG data with a cell phone camera, messaging app, or via a verbal report. These methods require EMTs to have high ECG interpretation abilities, and the use of an unencrypted mobile device can compromise personal information. This leads to issues regarding Health Insurance Portability and Accountability Act data breaches [30]. A safe and easy way to transfer information from devices, mobile devices, or a connected app is needed.

For the treatment of CVD, an effective and efficient way to integrate prehospital care into in-hospital care should be sought to improve the overall quality of care. Simple 12-lead ECG measurement and easy transmission would not only contribute to the quality of prehospital emergency care but also improve hospital care. In the future, these technologies are expected to extend to patients’ homes, enabling 12-lead ECG management of CVD by the patients themselves.

### Limitations

This study had some limitations. First, this was a simulation-based study, and the driving course and speed of the ambulance were limited to within the hospital area. The ambulance was only able to reach 30 km/h. Furthermore, our simulated patient was a healthy adult man. Second, the study location was in Seoul, where both LTE and 5G networks are well-implemented, and the average age of participants was 36 years. Therefore, transmission-related problems and bias may have occurred in other areas related to the use of mobile devices and app manipulation. Third, because only one device was used, it is difficult to represent the characteristics of other devices used in the prehospital environment. Environmental factors such as humidity can affect the touch sensitivity function of tablets and can cause recognition problems in the actual hospital environment. Fourth, this study collected 12-lead ECG data but only assessed time intervals and SUS survey responses as outcomes, without a comparison of the quality and accuracy of the two different ECG devices. Finally, although many studies have reported that reduction of total ischemic time may improve short- and long-term mortality, and reduce complications such as heart failure [31-33], this study only examined one component of STEMI treatment. Therefore, further research on the emergency medical system is needed.

### Conclusions

The performance and transmission of the 12-lead ECG examination using a patchy-type 12-lead ECG is faster than those when using the conventional device during ambulance transport. With this additional time, EMTs can provide more care to patients and transport patients more rapidly, which may help to reduce the symptoms-to-balloon time for patients with ACS.

## Abbreviations

ACS: acute coronary syndrome
C-ECG: conventional electrocardiogram
CVD: cardiovascular disease
ECG: electrocardiogram
EMS: emergency medical service
EMT: emergency medical technician
LTE: long-term evolution
P12ECG: prehospital 12-lead electrocardiogram
P-ECG: patchy-type wireless electrocardiogram
STEMI: ST-segment elevation myocardial infarction
SUS: System Usability Scale
